# Tracheal microbial populations in horses with moderate asthma

**DOI:** 10.1111/jvim.15707

**Published:** 2020-01-27

**Authors:** Estelle Manguin, Elizabeth Pépin, Roxane Boivin, Mathilde Leclere

**Affiliations:** ^1^ Department of Clinical Sciences, Faculty of Veterinary Medicine Université de Montréal Quebec Canada

**Keywords:** bacteria, dysbiosis, inflammatory airway disease, microbiota

## Abstract

**Background:**

There are limited data on potential dysbiosis of the airway microbiota in horses with asthma.

**Hypothesis/Objectives:**

We hypothesized that the respiratory microbiota of horses with moderate asthma is altered. Our objectives were (a) to quantify tracheal bacterial populations using culture and qPCR, (2) to compare aerobic culture and qPCR, and (c) to correlate bacterial populations with bronchoalveolar lavage fluid (BALF) cytology.

**Animals:**

Eighteen horses with moderate asthma from a hospital population and 10 controls.

**Methods:**

Prospective case‐control study. Aerobic culture was performed on tracheal aspirates, and streptococci, *Pasteurella multocida*, *Chlamydophila* spp., *Mycoplasma* spp., as well as 16S (bacterial) and 18S (fungal) rRNA subunits were quantified by qPCR.

**Results:**

Potential pathogens such as *Streptococcus* spp., *Actinobacillus* spp., and *Pasteurellaceae* were isolated from 8, 5, and 6 horses with asthma and 3, 0, and 2 controls, respectively. There was a positive correlation between *Streptococcus* spp. DNA and 16S rRNA gene (r ≥ 0.7, *P* ≤ 0.02 in both groups), but the overall bacterial load (16S) was lower in asthma (1.5 ± 1.3 versus 2.5 ± 0.8 × 10^4^ copy/μL, *P* < 0.05). There was no association between microbial populations and clinical signs, tracheal mucus or BALF inflammation.

**Conclusions and Clinical Importance:**

This study does not support that bacterial overgrowth is a common feature of chronic moderate asthma in horses. Lower bacterial load could suggest dysbiosis of the lower airways, either as a consequence of chronic inflammation or previous treatments, or as a perpetuating factor of inflammation.

**Abbreviations**
ACVIM: American College of Veterinary Internal Medicine
BALF: bronchoalveolar lavage fluid
CDEVQ: Provincial Veterinary Diagnostic and Epidemiology Laboratory
CFU: colony forming units
DNA: deoxyribonucleic acid
PBS: phosphate buffered saline
qPCR: quantitative polymerase chain reaction
rpm: revolutions per minute
rRNA: ribosomal ribonucleic acid
spp.: species pluralis
subsp.: subspecies

## 1 INTRODUCTION

Mild and moderate asthma, formerly “inflammatory airway disease,” is characterized by airway inflammation and airflow limitation. Clinical signs include cough, nasal discharge, and poor performance. Diagnosis is based on the presence of compatible clinical signs and documentation of pulmonary inflammation with bronchoalveolar lavage fluid (BALF) cytology, or airflow obstruction with lung function testing.^1^ In the absence of clear criteria to distinguish between mild and moderate, the terms “moderate asthma” is used here for clarity. Almost 15% of adult horses have severe asthma (heaves) and while the prevalence of moderate asthma is unknown, there is indirect evidence that close to 50% of horses will have at least 1 episode of cough during their life.^2^

The etiology of asthma is mainly immune and environmental, which is supported in part by the positive response of affected horses to corticosteroid administration and decreased exposure to hay.^1^ The role of bacterial infection or colonization in the development of asthma in horses is uncertain, but it could be a contributing factor to exacerbations of clinical signs. For example, there is an association between tracheal bacterial count and cough,^3^ and between tracheal mucus and bacteria isolated from tracheal aspirates in racehorses.^4^, ^5^ The pulmonary microbiome of horses with asthma is different from that of healthy horses, and the pulmonary microbiome is influenced both by the presence of the disease and by the environment.^6^, ^7^ In human asthma, bacteria have received less attention than viruses for their role in clinical exacerbations, but infections with *Haemophilus influenzae*, *Moraxella catarrhalis*, and *Streptococcus pneumoniae* are now recognized as opportunistic pathogens associated with acute asthma exacerbations.^8^, ^9^, ^10^ Furthermore, changes in the composition of the lung microbiome are associated with bronchial hyperresponsiveness^11^ and with corticosteroid resistance.^12^

In clinical cases, tracheal aspirates are often performed with BALF analysis in horses suspected of moderate asthma, but the interpretation of tracheal bacterial culture is complicated by the bacterial diversity in healthy horses.^13^ Moreover, culture‐independent methods (PCR and sequencing) have highlighted that standard bacterial culture allows for the identification of only small subsets of bacteria.^14^ This study proposes to document microbial species found in tracheal aspirates collected from horses with and without moderate asthma by conventional methods (aerobic culture) and molecular methods (quantitative polymerase chain reaction [qPCR]). The underlying hypothesis is that dysbiosis in the microbial populations could contribute to sustained pulmonary inflammation and possibly affect response to treatment. More specifically, we hypothesized that the respiratory microbiota of horses with moderate asthma is altered, with more tracheal bacteria than in controls, and that there is a link between lower airway bacterial populations and pulmonary inflammation. The aims of this study were (a) to quantify bacterial populations using bacterial culture and qPCR, (b) to compare aerobic culture and qPCR, and (c) to correlate bacterial populations with measures of BALF inflammation.

## 2 MATERIALS AND METHODS

### 2.1 Horses

To account for the difficulty of recruiting healthy controls, sample sizes were calculated for a 2 to 1 ratio for diseased versus healthy horses. Sample sizes were calculated to achieve a power of 80% with a 2‐sample *t* test and an alpha level of 5%. For BALF neutrophil percentages estimated, mean and range for controls was 5% (3%‐7%) and 20% (15%‐50%) for the asthma group and the power analysis suggested 5 controls and 11 asthmatic horses. Bacterial counts were more difficult to estimate based on the literature^4^ and the fact that they are unlikely to be normally distributed. Conservative estimates suggested that 5 controls and 9 asthmatic horses would be sufficient, but to account for sampling variation, we aimed to recruit 10 controls and 20 asthmatic horses, and were able to recruit 10 and 18, respectively. Horses presented to the equine hospital of the University of Montreal between January 2015 and November 2016 were enrolled with owners’ consent. Horses with moderate asthma had to meet the following criteria: (a) 1 or more clinical signs compatible with chronic respiratory disease (cough, nasal discharge, decreased performance) and (b) evidence of BALF inflammation (neutrophils ≥10%, mast cells ≥2%, eosinophils ≥1%). Horses with signs of systemic illness or other causes of decreased performance were excluded from the asthma group. Thoracic radiographs, dynamic endoscopy, or cardiac evaluation were performed when deemed indicated by the attending clinician. During the same period, control horses with no history of respiratory disease were recruited and examined under the same conditions. They had no signs of respiratory disease on physical examination, including rebreathing bag examination, and no evidence of inflammation in their BALF (neutrophils <10%, mast cell <2%, eosinophils <1%) or on complete blood count. The experimental protocol was performed in accordance with the Canadian Council on Animal Care guidelines and was approved by the Animal Care Committee of the Faculty of Veterinary Medicine of the University of Montreal (protocol #14‐Rech‐1765).

### 2.2 Upper airway endoscopy and mucus scoring

Upper airway endoscopy was performed under sedation (xylazine, Rompum, Bayer, Mississauga, ON, Canada) 0.4 to 0.5 mg/kg IV or detomidine (Dormosedan, Zoetis, Parsippany, New Jersey) 0.008 to 0.1 mg/kg IV, and butorphanol (Torbugesic, Zoetis, Florham Park, New Jersey) 0.01 to 0.02 mg/kg IV) with a 1.6 m fiber‐optic videoendoscope (Olympus, GIF‐H180, Olympus Canada Inc., Richmond Hill, ON, Canada). Tracheal mucus accumulation was scored from 0 to 5, as previously described,^15^ with 0 corresponding to no mucus, 1 to little mucus accumulation in small blobs, 2 to moderate mucus accumulation, 3 to marked, stream forming mucus blobs, 4 to large, pool‐forming accumulation, and 5 to extreme accumulation of mucus. A score greater than 2 was considered abnormal.^1^

### 2.3 Tracheal aspirates

Tracheal aspirates were collected via a triple guarded endoscopic catheter (Triple stage catheter, MILA Endoscopic Microbiology Aspiration Catheter EMAC800, Mila International, Florence, Kentucky), approximately 10 cm proximally to the main carina. Twenty milliliter of sterile isotonic saline were infused and quickly aspirated. All samples were put immediately on ice and separated for bacterial and qPCR analysis. The samples for culture were submitted to the CDEVQ laboratory (Provincial Veterinary Diagnostic and Epidemiology Laboratory) within 1 hour and the samples for qPCR were processed and frozen at −20°C within 1 hour.

### 2.4 Bronchoalveolar lavage

Bronchoalveolar lavage fluid was collected after the tracheal aspirate, as previously described.^16^ The videoendoscope was advanced into the right principal bronchus until it wedged into a smaller bronchus while infusing 60 to 120 mL of lidocaine 0.5%. Two boluses of 250 mL of sterile prewarmed isotonic saline (0.9% NaCl) were infused and quickly reaspirated. Samples were processed within 1 hour. Smears were prepared by centrifugation (Cytospin model II, Shandon Southern Instruments, Sewickley, Pennsylvania) and stained with a modified Wright‐Giemsa. Differential count was performed on 400 nucleated cells, excluding epithelial cells, by a board‐certified clinical pathologist.

### 2.5 Aerobic culture on tracheal aspirates

Standard aerobic bacterial culture was performed on blood agar plates. For each sample, quantification was estimated with 2 techniques: (a) direct plating (spread‐plate method, reported as colony forming units per ml [CFU/mL]), and (b) postcentrifugation (quadrant streaking method, reported as scores). Ten microliters of tracheal aspirate were directly spread on the plate (direct plating) and incubated at 37°C for approximately 24 to 48 hours before the colonies were counted. In order to increase the odds of isolating bacteria present in low concentrations, the rest of the sample was centrifuged and then plated according to the quadrant streaking method. Briefly, after a 24 to 48 hour incubation period, colonies were counted and reported semiquantitatively using a 0 to 5 system: a score of 0 represents no growth; 1 indicates that there is 1 colony in the first quadrant; 2 indicates 2 to 4 colonies in the first quadrant; 3 indicates 5 or more colonies in the first quadrant; 4 indicates that there are colonies in the second quadrant; and 5 indicates that there are colonies in the third quadrant. Culture was determined to be negative after 5 days with no growth. A direct smear was done using the pellet from the centrifuged sample and stained with the standard Gram's staining.

### 2.6 Quantitative PCR on tracheal aspirates

One milliliter of tracheal aspirate was centrifuged for 30 minutes at 13 200 rpm (rpm). Supernatant was removed and the pellet was resuspended in 100 μL of phosphate buffered saline (PBS). Deoxyribonucleic acid (DNA) extraction was performed with the QIAGEN DNeasy Kit (Qiagen, Toronto, ON, Canada) on samples following manufacturer's instructions with minor modifications. An enzymatic lysis buffer was used for pretreatment instead of mechanical lysis, and the elution volume was 50 μL instead of 100 μL. Quantitative PCR were performed for *Streptococcus* spp., *Streptococcus equi* subspecies (subsp.) *zooepidemicus*, *Streptococcus equi* subsp. *equi*, *Streptococcus pneumoniae*, *Pasteurella multocida*, *Chlamydophila* spp., *Mycoplasma* spp., 16S rRNA gene (gene coding for the 16S subunit of bacterial ribosomal RNA), and 18S rRNA gene (gene coding for the 18S subunit of fungal ribosomal RNA). Primers are presented in Table 1, with their optimal cycle conditions. Most of them were designed manually from a specific gene sequence and all primer sets were tested for sensitivity, optimal annealing temperature, and efficiency. For each target, high‐quality DNA was extracted from colonies provided by the CDEVQ laboratory and was used to build standard curves with an efficiency ≥90% and an *R* ^2^ ≥ 0.998. Primers for *Actinobacillus equuli*, *suis*, *ligneresii*, and *pleuropneumoniae* were also designed but satisfactory standard curves could not be attained. All qPCR tests were carried out in duplicates (except for *Streptococcus equi* subsp. *equi* due to the limited amount of tracheal aspirate available) and means of the duplicates were used for analysis. Quantitative PCR reactions were performed in 20 μL volume with 5 μM primers, using the Rotor‐Gene RG3000 (Corbett Research, Mortlake, Australia) with the QuantiTect SYBR Green PCR Kit (Qiagen, Toronto, ON, Canada). Negative controls included all elements of the reaction mixture except DNA and were performed with every analysis. No amplified DNA product was detected in the negative controls. Copy numbers per microliter were calculated relative to the appropriate standard curve. Samples falling below the detection limit were assigned the detection limit (cycle threshold = 40).

**Table 1:** Quantitative PCR primers and cycling conditions for the bacteria and ribosomal genes targeted

	Forward primer (5′‐3′)	Reverse primer (5′‐3′)	Cycle conditions
*Streptococcus* spp.	GTTCGATTTCATCACGTTGAA	ACAGTTGCTTCAGGACGTA	95°C—10 min, 95°C—10 s, 54°C—15 s, 72°C—15 s
*Streptococcus equi* subsp. *zooepidemicus*	CAGCATTCCTGCTGACATTCGTCAGG	CTGACCAGCCTTATTCACAACCAGCC	95°C—10 min, 95°C—15 s, 68°C—15 s, 72°C—20 s
*Streptococcus equi* subsp. *equi*	CGGATACGGTGATGTTAAAGA	CTCTCTGTCACCATGTCCT	95°C—10 min, 95°C—10 s, 52°C—15 s, 72°C—15 s
*Streptococcus pneumoniae*	ACCGATGGCGCTGGTCTTCAT	GATGTCTTATAGCCACCTTGACC	95°C—10 min, 95°C—15 s, 60°C—15 s, 72°C—20 s
*Pasteurella multocida*	GGGCTTGTCGGTAGTCTTT	CGGCAAATAACAATAAGCTGAGTA	95°C—10 min, 95°C—15 s, 55°C—15 s, 72°C—15 s
*Chlamydophila* spp.	CGCTCTCTCCTTACAAGCCT	GCTAATGGCGTCACACCAAG	95°C—10 min, 95°C—10 s, 55°C—15 s, 72°C—15 s
*Mycoplasma* spp.	GGTTAACAGAGTGACAGATGGTGC	CCCCACTCGTAAGAGGCATGATG	95°C—10 min, 95°C—15 s, 60°C—15 s, 72°C—15 s
16S rRNA gene	ACACGGCCCAGACTCCTAC	TATTACCGCGGCTGCTGGC	95°C—10 min, 95°C—10 s, 59°C—15 s, 72°C—20 s
18S rRNA gene	AAAGGAATTGACGGAAGGGCA	TCACAGACCTGTTATTGCCTC	95°C—10 min, 95°C—10 s, 59°C—15 s, 72°C—20 s

Abbreviations: spp., species pluralis; subsp., subspecies.

### 2.7 Statistical analysis

Statistical analyses were performed using commercial software (GraphPad Prism 8.0.1 and, for Cohen's *kappa* coefficients, SAS, version 9.3, SAS Institute Inc, Cary, North Carolina). Normality was assessed by visual inspection of the data and the Kolmogorov‐Smirnov test. Data not normally distributed were log transformed or compared with nonparametric tests, when appropriate. Age, log transformed quantitative culture results (log10 CFU/ml + 1), log transformed qPCR results (log 10) were compared using an unpaired *t* test with Welch's correction. Tracheal mucus scores, semiquantitative bacterial scores, and BALF cytology were compared using the Mann‐Whitney test. Data were also analyzed without horses with a history of antimicrobials or corticosteroids administration (see *Previous medication*), and, within the asthma group, between horses with and without prior treatment. Proportions (sex, presence or absence of bacteria, clinical signs, BALF inflammation, or mucus score >2) were compared with the Fisher's exact test. Agreement between culture and qPCR for *Streptococcus* spp. was evaluated with binary classification (presence/absence), with the Cohen's *kappa* coefficient. Pearson's correlations on qPCR log transformed data between results for the 16S rRNA gene and specific bacteria were performed. For all statistical tests, values of *P* < 0.05 were considered significant.

## 3 RESULTS

### 3.1 Horses

**Figure 1:**
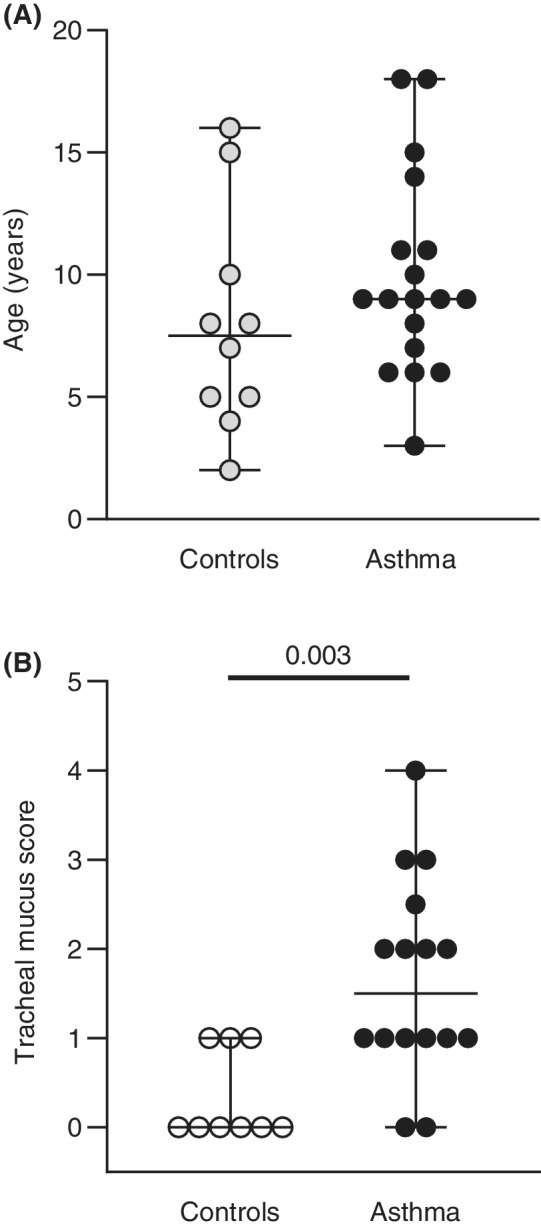
Age and tracheal mucus scores. A, Age of 10 control horses (white circles) and 18 horses with moderate asthma (black circles). Lines represent the median and range. B, Mucus scores of 9 control horses (white circles) and 17 horses with moderate asthma (black circles). Lines represent the median and range

Horses with asthma included 7 mares and 11 geldings, aged from 3 to 18 years old (mean ± SD: 9.89 ± 4.10 years). The control group included 4 mares and 6 geldings, aged from 2 to 16 years old (8.00 ± 4.57 years). There was no significant difference between groups for age (*P* = 0.29, Figure 1A) and sex (*P* > 0.9). Nine horses with asthma and 5 controls were Quarter Horses and associated breeds. Other breeds included Warmbloods (3 in each group), Shires, Standardbreds, Thoroughbreds, Andalusians, Canadians, and mixed breeds. All horses with asthma presented with compatible clinical signs, namely cough (n = 17), nasal discharge (n = 11), and poor performance (n = 6). The 6 horses with a complaint of poor performance had at least 1 other sign suggestive of respiratory disease. None of the controls had a history or clinical signs of respiratory disease. One control horse presented for poor performance was diagnosed with myocarditis. Tracheal mucus scores were available for 17 of the 18 horses with asthma and 9 of the 10 controls. The scores were higher in the asthma group (*P* = 0.003) (Figure 1B).

### 3.2 Previous medication

Four horses with moderate asthma had received antimicrobials (ceftiofur or trimethoprim‐sulfadiazine) 1 week (2 horses) and 4 weeks (2 horses) prior to presentation. Another 5 horses had received ceftiofur or trimethoprim‐sulfadiazine between 1 and 3 months prior to presentation. None were receiving antimicrobials at the time of presentation. Nine horses with moderate asthma had received systemic or inhaled corticosteroids (dexamethasone, prednisolone, fluticasone) in the 3 months prior to presentation, 1 of them in the 2 weeks prior to presentation and 2 others during the week of presentation. Other treatments received prior to presentation included bronchodilators, expectorants, antihistamines, and nonsteroidal anti‐inflammatory drugs. One control horse (with myocarditis) was empirically treated with corticosteroids (prednisolone) in the 2 weeks prior to presentation, due to his poor performance. Analyses were done with and without this horse, with no changes in results. He was therefore kept in the final analysis.

### 3.3 Bronchoalveolar lavage cytology

**Figure 2:**
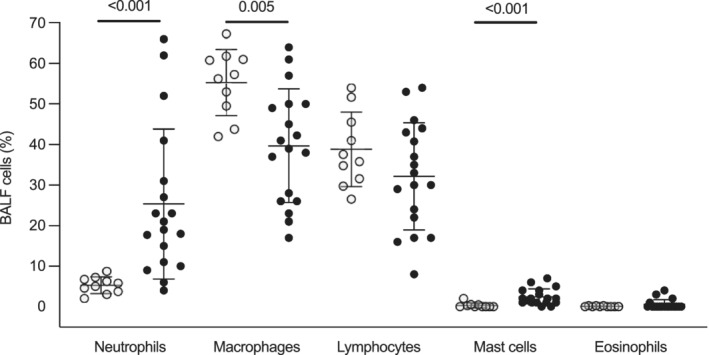
Bronchoalveolar lavage cytology. Percentages of neutrophils, macrophages, lymphocytes, mast cells, and eosinophils in the bronchoalveolar lavage fluids of 10 control horses (white circles) and 18 horses with moderate asthma (black circles). Lines represent the mean and SD

Bronchoalveolar lavage fluid neutrophil and mast cell percentages were significantly higher (*P* < 0.001), and macrophages were significantly lower (*P* = 0.01) in horses with asthma (Figure 2). There was no significant difference between groups for eosinophils and lymphocytes.

### 3.4 Aerobic culture of tracheal aspirates

**Figure 3:**
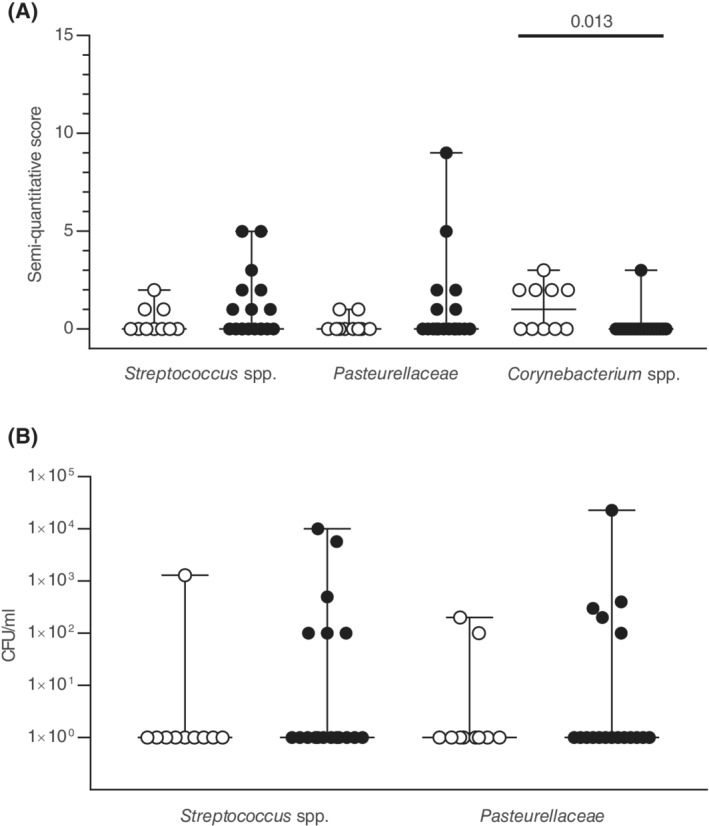
Tracheal aspirates aerobic culture. Aerobic bacterial culture results for 10 control horses (white circles) and 18 horses with moderate asthma (black circles). Lines represent the median and range. A, Semiquantitative scores from the quadrant streaking method. The scores are cumulative and can therefore be greater than 5. *Pasteurellaceae* include *Actinobacillus* spp., *Pasteurella* spp., and others *Pasteurellaceae*. *Streptococcus* spp. include all streptococci isolated. B, Colony forming units (CFU) after direct plating of 10 μL of tracheal aspirate

Forty‐four percent (8/18) of tracheal aspirates from horses with asthma and 10% (1/10) of controls yielded no growth (not significantly different between groups, *P* = 0.10). This takes into account the growth of any bacteria, including potential “contaminants” such as *Corynebacterium* spp. and *Staphylococcus* spp., as well as unidentified bacilli growing in small amounts. When *Corynebacterium* spp., often described as commensal bacteria of the airways, were analyzed separately, they were significantly more common in control horses (Figure 3A, *P* = 0.01). This remained true when horses who had received antimicrobials or corticosteroids were removed from analysis (*P* = 0.03 and 0.03, respectively). *Streptococcus* spp. (including *alpha* and *nonhemolytic Streptococcus*) were isolated from 8 horses with asthma and 3 controls (semiquantitative scores, Figure 3A). *Actinobacillus* spp. were isolated from 5 horses with asthma and 0 controls and *Pasteurellaceae* (including *Actinobacillus* spp. and *Pasteurella* spp.) from 6 horses with asthma and 2 controls (Figure 3A). The proportion of cultures positive for *Streptococcus* spp. and *Pasteurellaceae* were not significantly different from controls (Figure 3A, all *P* > 0.1). Results were similar when horses who had received antimicrobials or corticosteroids were removed from analysis, except for the greater proportion of horses with asthma that were positive for *Actinobacillus* spp. (*P* = 0.03 for both). Within the group of horses with asthma, there were no significant differences between horses that had received prior treatments or not (*P* > 0.2). Other bacteria occasionally isolated included *Bacillus* spp., *Enterobacter* spp., *Nocardia* spp., and *Enterococcus* spp. Colony forming units per milliliter and cumulative semiquantitative scores for *Streptococcus* spp. or *Pasteurellaceae* were not significantly different between groups (Figure 3B, *P* ≥ 0.1). Gram‐positive or Gram‐negative bacteria were observed on 8 direct smears, and among those, 7 had a positive growth that were consistent with the Gram results. There was no association between a positive culture of *Streptococcus* spp. or *Pasteurellaceae* with the presence of clinical signs, tracheal mucus, or neutrophilic BALF (*P* > 0.2). Table 2 summarizes the proportion of samples positive by direct smears and semiquantitative aerobic culture.

**Table 2:** Summary of the proportion of positive direct smears, semiquantitative aerobic culture and qPCR on tracheal aspirates in control horses and horses with moderate asthma

		Controls % of positive (n = 10)	Asthma % of positive (n = 18)	*P* value
Direct smear	Bacteria observed (any)	50	17	0.1
Aerobic culture	Growth (any)	90	56	0.1
	*Corynebacterium* spp.	50	6	0.013
	*Streptococcus* spp.	30	44	>0.1
	*Actinobacillus* spp.	0	28	>0.1
	*Pasteurellaceae*	20	33	>0.1
qPCR	16S rRNA gene	100	100	‐
	18S rRNA gene	100	100	‐
	*Streptococcus* spp.	40	61	>0.1
	*Pasteurella multocida*	20	11	>0.1
	*Chlamydophila* spp.	60	44	>0.1
	*Mycoplasma* spp.	0	6	>0.1

### 3.5 Real‐time PCR of tracheal aspirates

**Figure 4:**
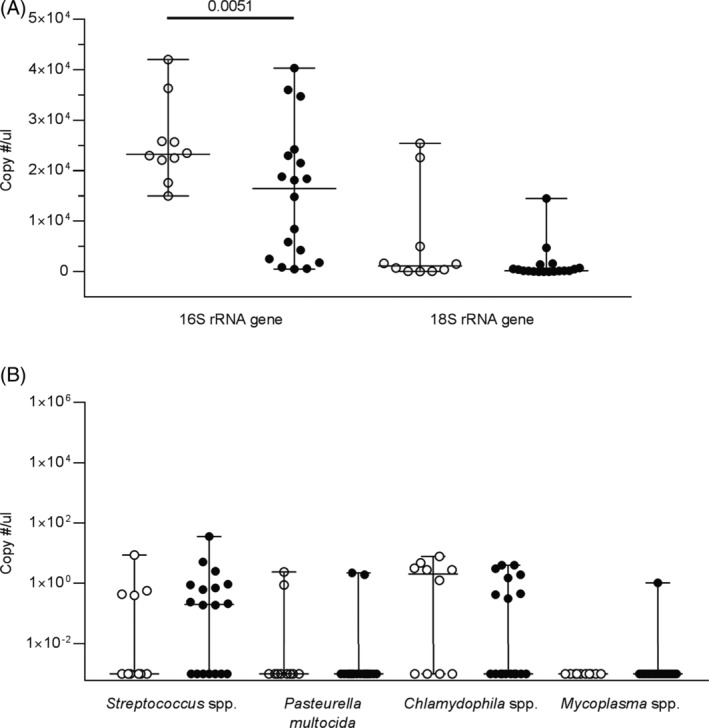
Tracheal aspirate qPCR. Real‐time PCR results for 10 control horses (white circles) and 18 horses with moderate asthma (black circles). Lines represent the median and range. A, Copy number per microliter for 16S rRNA gene and 18S rRNA gene. B, Copy number per microliter for *Streptococcus* spp., *Pasteurella multocida*, *Chlamydophila* spp., *Mycoplasma* spp

All tracheal aspirates were positive for the 16S and 18S rRNA genes, with overall higher concentrations for 16S than for 18S, and higher concentrations of the 16S rRNA gene in controls than in horses with asthma (Figure 4A, *P* = 0.01). This remained true when horses who had received antimicrobials were removed from analysis (*P* < 0.05) but not when horses with corticosteroids were removed (*P* = 0.06). Within horses with asthma, there was no significant differences between horses with and without prior treatments (*P* > 0.6). *Streptococcus* spp. and *Chlamydophila* spp. were the 2 bacteria most commonly identified, with no differences between groups (Figure 4B). The quantification of specific Streptococci (*equi*, *zooepidemicus*, and *pneumoniae*) was positive only for *Streptococcus pneumoniae* in a control horse. *Mycoplasma* spp. and *Pasteurella multocida* were also only detected occasionally in each group (Figure 4B). The proportion of samples positive by qPCR are summarized in Table 2.

There was a strong positive correlation between 16S qPCR results and *Streptococcus* spp. (r = 0.72, *P* = 0.02 for controls, r = 0.69, *P* = 0.002 for horses with asthma), and between 16S and 18S results, but only in the control group (r = 0.83, *P* = 0.003). There was an overall poor concordance between positive *Streptococcus* spp. qPCR results and growth (kappa = 0.16, *P* = 0.18). In fact, qPCR and culture agreed on the presence or absence of *Streptococcus* spp. in 57% of the cases (16/28), but in 7 cases some *Streptococcus* spp. were detected by PCR yet not by culture, and in 3 samples, *Streptococcus* spp. grew in culture (in a small amount) but remained below the detection level of qPCR. The presence or absence of *Streptococcus* spp. or *Chlamydophila* spp. was not associated with asthma, or specific clinical signs, high mucus scores and BALF inflammation (all *P* > 0.25).

## 4 DISCUSSION

The results from this study do not support our hypothesis that horses with moderate asthma have greater bacterial loads, or that qPCR would increase the number of horses for which an infectious component would be suspected. There was a lower burden in horses with asthma (based on 16S rRNA gene quantification), with more than twice as many samples with no growth in that group, and *Corynebacterium* spp. was significantly more common in control horses. The lower bacterial load and less frequent commensal bacteria (*Corynebacterium* spp.) in our population of horses with asthma suggests that the respiratory bacterial microbiota might be altered in moderate asthma, but that bacterial overgrowth is not a consistent feature in these horses.

### 4.1 Airway dysbiosis in equine and human asthma

These results are in contrast with studies performed in racehorses where bacteria, mainly streptococci and *Pasteurellaceae*, are associated with signs of respiratory disease,^3^ tracheal mucus,^4^, ^17^ and tracheal inflammation.^18^, ^19^ These previous studies mostly focused on young racehorses that are more likely to have viral and bacterial infections than older horses, as well as to have contamination from the environment during strenuous exercise. The horses in the current study were on average ≥8 years old (ie, older than racehorses) and, to be included, their condition had to have a certain chronicity, as per the American College of Veterinary Internal Medicine (ACVIM) guidelines.^1^ These criteria are rarely met on the racetrack, where horses tend to be examined shortly after they develop a cough, and sometimes in the absence of clinical signs, and where inflammation is also often based on tracheal cytology instead of BALF cytology. Furthermore, horses in the current study were likely to have been treated for their respiratory condition, including with antimicrobials and corticosteroids in the months before presentation.

In human asthma, in addition to viral infections, specific bacterial pathogens are now recognized as being associated with exacerbations,^8^, ^9^, ^10^ but few studies have documented an increase in overall bacterial load in asthmatic patients.^11^ Evidence of dysbiosis is mostly supported by studies using next‐generation sequencing showing differences in the relative abundance of the bacteria present (commensal and potential pathogens). This has been documented in children and adults with asthma, and dysbiosis is now associated not only with asthma prevalence and acute exacerbation, but also with asthma severity and airway hyperresponsiveness.^11^, ^12^, ^20^, ^21^ Dysbiosis of the airways was also described recently using next‐generation sequencing in horses with moderate and severe asthma,^6^, ^7^ using inclusion criteria in line with the current ACVIM recommendations. Tracheal microbiota was altered in horses with moderate asthma, with a greater relative abundance of the genus *Streptococcus* spp.,^6^ and pulmonary microbiota was also altered in horses with severe asthma, but without overrepresentation of specific pathogens in bronchoalveolar lavages.^7^ Neither studies attempted to quantify the absolute amount of specific pathogens. At this point, it is still unclear as whether bacterial dysbiosis in asthma should be considered a causative factor of asthma exacerbation, a perpetuating factor of inflammation, or a consequence of chronic inflammation. Also, because of the delay between the onset or worsening of clinical signs and referral, our study does not rule out overgrowth of bacteria (all populations or specific pathogens) but sampling would have to be done earlier during an exacerbation to better address this question.

*Streptococcus* spp. were the most common bacteria isolated in culture and detected by qPCR, but few were from the *Streptococcus zooepidemicus* species. In contrast, *Streptococcus zooepidemicus* was isolated from 5.3% to 6.4% and 16% to 37.7% of horses in previous studies on racehorses.^4^, ^17^ Other studies in nonracehorses have found lower prevalences of *Streptococcus zooepidemicus*, including one with no positive growth in 24 tracheal aspirates done in healthy horses before and after transportation.^13^ With the low prevalence in our population, it is not surprising that we did not find an association with disease status or clinical signs like others have in different populations.^4^, ^17^

### 4.2 The role of airway microbiota

Because controls had higher 16S rRNA gene loads and *Corynebacterium* spp., we hypothesize that bacteria present in small amounts, including bacteria often referred to as “contaminants” by diagnostic laboratories (*Corynebacterium* spp., *Staphylococcus* spp., *Bacillus* spp., unidentified Gram negatives), should probably be considered part of the microbial population of healthy equine airways. In humans, colonization of the nasopharynx by *Staphylococcus* spp. and *Corynebacterium* spp. is considered normal in healthy young children,^22^ and in vitro, *Corynebacterium accolens* shows antagonistic interactions with *Streptococcus pneumoniae*, a common pathogen in humans.^23^ Thus, it is suggested that commensal bacteria could act against potential pathogens and help maintain a normal airway environment.

### 4.3 Selection of bacteria for qPCR

In addition to *Streptococcus zooepidemicus*,^3^, ^4^, ^17^ other species of the *Streptococcus* genus have been associated with cough and respiratory inflammation, most importantly *Streptococcus pneumoniae*.^3^, ^4^, ^18^, ^19^ *Streptococcus suis* and *Streptococcus sanguis* have also been isolated in racehorses with clinical signs.^3^ As all species could not be targeted individually, we elected to build primers that could detect multiple species of streptococci, in addition to the primers specific for *Streptococcus zooepidemicus*, *equi*, and *pneumoniae*. Other bacteria targeted by qPCR in this study included *Pasteurella multocida* and *Mycoplasma* spp. *Pasteurella* spp. were associated with cough and *Mycoplasma* spp. with tracheal mucus in horses.^3^, ^4^ *Mycoplasma* spp. are also found in children with asthma.^24^ *Chlamydophila* spp., another atypical bacteria associated with asthma exacerbation in humans,^25^ were detected by immunohistochemistry more frequently in the bronchial epithelial cells of asthmatic horses.^26^ It is also important to realize that several bacteria such as *Chamydophila* spp. and *Mycoplasma* spp. do not grow or are not readily identified on standard culture, which reinforces the relevance of culture independent techniques such as PCR. Finally, *Actinobacillus* spp. (from the *Pasteurellaceae* family) are also associated with increased tracheal mucus in racehorses.^17^ In the current study *Actinobacillus* spp. were isolated by culture in the asthma group only. Unfortunately, despite numerous attempts and different primers tested, standard curves of adequate accuracy could not be attained.

### 4.4 Potential confounding factors

Investigations based on clinical cases come with conditions that cannot be controlled as tightly as in laboratory research settings. One potential confounding factor is the fact that many horses in our study had received corticosteroids or antimicrobials in the months prior to presentation, and both medications have the potential to affect airway microbiota. Specifically, dexamethasone induced changes in the tracheal microbiota of healthy and asthma‐affected horses, with a relative increase in the abundance of certain genera of bacteria (*Peptostreptococcus*, *Porphyromonas*, *Filifactor*, *Streptococcus*, *Porphyromonas*, *Parvimonas*, *Fusobacterium*, and *Bacteroides* spp.) but with a decrease in 1 operational taxonomic unit from the ubiquitous candidate Saccharibacteria phylum.^6^ The duration of the change in microbiota after cessation of antimicrobial treatment was not investigated. However, in another study, the addition of antibiotics (ceftiofur) to a standard treatment with corticosteroids improved clinical scores but did not change the amount or type of bacteria cultured from their tracheal aspirates.^27^ This suggests that previous treatment with antimicrobials (mostly with ceftiofur and trimethoprim‐sulfadiazine) are less likely to be a major confounding factor in our study. As horses were not on antimicrobials at the time of the visit, it is unlikely to have influenced directly the results of the culture and PCR, but that could have decreased the prevalence of secondary bacterial infections. Our data suggest that prior antimicrobial administration was not the cause of lower bacterial load in horses with asthma, with the possible exception of *Actinobacillus*, but interpretation is complicated by the fact that there is an overlap in the horses that received antimicrobials and corticosteroids in the asthma group (9 horses for each, but 7 received both, usually not at the same time). In addition, even in a subset of patients with a high positive culture or PCR, bacteria were not necessarily associated with tracheal mucus or clinical signs pointing toward bacterial overgrowth. Finally, mucus can decrease DNA extraction efficacy and could have contributed to the lower bacterial load (16S rRNA gene) in horses with asthma. Tracheal mucus should not have affected culture and therefore it is not a major confounding factor.

In conclusion, this study does not support that bacterial overgrowth is part of moderate equine asthma as defined by the current ACVIM consensus statement, at least in the chronic phase. However, lower 16S rRNA gene and commensal bacteria in the asthma group support the presence of lower airway dysbiosis. We cannot conclude from these data whether dysbiosis results from chronic inflammation or the treatments that these horses received. There was, however, a strong positive correlation between *Streptococcus* detected by PCR and 16S rRNA gene, suggesting that when bacteria are present, they are more likely to be of the *Streptococcus* genus. We can also conclude that large numbers of *Streptococcus zooepidemicus* isolated on tracheal aspirates should not be considered normal in healthy and asthmatic horses.

## CONFLICT OF INTEREST DECLARATION

Authors declare no conflict of interest.

## OFF‐LABEL ANTIMICROBIAL DECLARATION

Authors declare no off‐label use of antimicrobials.

## INSTITUTIONAL ANIMAL CARE AND USE COMMITTEE (IACUC) OR OTHER APPROVAL DECLARATION

The experimental protocol was performed in accordance with the Canadian Council on Animal Care guidelines and was approved by the Animal Care Committee of the Faculty of Veterinary Medicine of the University of Montreal (protocol #14‐Rech‐1765). Informed client consent was obtained.

## HUMAN ETHICS APPROVAL DECLARATION

Authors declare human ethics approval was not needed for this study.
